# Maternal and infant outcomes in women with and without gestational diabetes mellitus in the COVID-19 era in China: Lessons learned

**DOI:** 10.3389/fendo.2022.982493

**Published:** 2022-11-22

**Authors:** Wei Zheng, Jia Wang, Kexin Zhang, Cheng Liu, Li Zhang, Xin Liang, Lirui Zhang, Yuru Ma, Ruihua Yang, Xianxian Yuan, Guanghui Li

**Affiliations:** ^1^ Division of Endocrinology and Metabolism, Department of Obstetrics, Beijing Obstetrics and Gynecology Hospital, Capital Medical University, Beijing, China; ^2^ Beijing Maternal and Child Health Care Hospital, Beijing, China

**Keywords:** the COVID-19 pandemic, lockdown, gestational diabetes mellitus, pregnancy outcome, offspring outcome

## Abstract

**Aims:**

The global COVID-19 pandemic has required a drastic transformation of prenatal care services. Whether the reformulation of the antenatal care systems affects maternal and infant outcomes remains unknown. Particularly, women with gestational diabetes mellitus (GDM) are among those who bear the greatest brunt. Thus, this study aimed to evaluate the impact of COVID-19 lockdown during late pregnancy on maternal and infant outcomes in women stratified by the GDM status in China.

**Study design:**

The participants were women who experienced the COVID-19 lockdown during late pregnancy (3185 in the 2020 cohort) or not (2540 in the 2019 cohort) that were derived from the Beijing Birth Cohort Study. Maternal metabolic indicators, neonatal outcomes, and infant anthropometrics at 12 months of age were compared between the two cohorts, stratified by the GDM status.

**Results:**

Participants who experienced COVID-19 lockdown in late pregnancy showed lower gestational weight gain than those in the control cohort. Nevertheless, they displayed a worse metabolic profile. COVID-19 lockdown during pregnancy was associated with higher glycosylated hemoglobin (HbA1c) (β= 0.11, 95% CI = 0.05–0.16, q-value = 0.002) and lower high density lipoprotein cholesterol level (HDL-C) level (β=–0.09, 95% CI = –0.14 to –0.04, q-value = 0.004) in women with GDM, adjusted for potential confounders. In normoglycemic women, COVID-19 lockdown in late pregnancy was associated with higher fasting glucose level (β= 0.10, 95% CI = 0.08–0.12, q-value <0.0001), lower HDL-C level (β=–0.07, 95% CI = –0.08 to –0.04, q-value <0.0001), and increased risk of pregnancy-induced hypertension (adjusted OR=1.80, 95%CI=1.30–2.50, q-value=0.001). The fasting glucose level decreased less from early to late pregnancy in women who experienced COVID-19 lockdown than in the controls, regardless of the GDM status. The HDL-C has risen less with COVID-19 lockdown in the normoglycemic subgroup. In contrast, no significant differences regarding neonatal outcomes or infant weight were found between the two cohorts.

**Conclusion:**

Experiencing the COVID-19 lockdown in pregnancy was associated with worse maternal metabolic status but similar neonatal outcomes and infant weight.

## Introduction

The coronavirus disease 2019 (COVID-19) pandemic is rampant worldwide and has challenged the healthcare system (1). Emergency measures such as social distancing, reallocating medical resources, and adapting medical strategies have been implemented to curb the unprecedented crisis (2). These contingency strategies have disrupted the original order of medical services and brought difficulties to the health management of vulnerable populations such as pregnant women (3).

Cases of pneumonia with unknown causes emerged in Wuhan, China, in December 2019. Following the pandemic evolution and lockdown of Wuhan on 23 January 2020, the first-level public health emergency response was launched in many provinces, districts, and cities including Beijing in China. After more than three months of strict prevention and control, Beijing has changed the level of public health emergency response from first-level to second-level from 30 April 2020, and adjusted prevention and control strategies accordingly. Pregnant women with metabolic disorders are among those who bear the greatest brunt of the crisis (3). Gestational diabetes mellitus (GDM) is one of the most common pregnancy complications affecting about 14% of pregnant women (4), profoundly impacting the short-term and long-term health of both mothers and their offspring (5). While desirable glycemic control during pregnancy can reduce the risk of future type 2 diabetes and dyslipidemia in mothers (6), neonatal adiposity and childhood obesity in their offspring (7, 8), and thereby has important implications for breaking the intergenerational transmission of metabolic diseases. However, the unprecedented COVID-19 pandemic has posed challenges to regular prenatal check-ups during pregnancy and blood glucose monitoring for pregnant women with GDM (9).

In addition, pregnant women during the COVID-19 pandemic experienced heightened anxiety levels (10, 11). Restrictions during the COVID-19 pandemic, including social distancing, isolation, and home confinement, also substantially impacted dietary habits and physical activity (12, 13). The above factors may significantly influence both maternal and neonatal outcomes of pregnant women (7). A previous study by Ghesquière et al. has reported that the COVID-19 pandemic lockdown may result in poor glycemic control in women with GDM (14). However, there is a lack of data to comprehensively evaluate the impact of COVID-19 and the temporary measures on maternal and infant outcomes of women with and without GDM.

Therefore, this study aimed to examine the influence of COVID-19 lockdown during late pregnancy on the maternal and infant outcomes stratified by the maternal GDM status.

### Material and methodsStudy design and settings

The study population was selected from the ongoing Beijing Birth Cohort Study conducted in the Beijing Obstetrics and Gynecology Hospital (registration number ChiCTR2200058395). The trained researchers recruited singleton pregnant women without pre-gestational diabetes mellitus (PGDM), including type 1 diabetes and type 2 diabetes, chronic hypertension or cardiovascular diseases at their first visit to the hospital at 6-12 weeks gestation. We excluded twin pregnant women since their maternal metabolic status and neonatal outcomes differed from singleton pregnancies. Our sample size is not enough for subgroup analysis in twin pregnancies. The participants were followed monthly until delivery, and their offspring were followed until 12 monthsonths. In this study, we selected 3029 pregnant women who received a 75 g oral glucose tolerance test (OGTT) for GDM diagnosis at 24–28 weeks of gestation between 23 January 2020 (the date of the lockdown of Wuhan and the implementation of first-level public health emergency response in Beijing) and 31 July 2020 and delivered during this period as the exposed study population. Accordingly, 3582 women who received the OGTT and deliver in the same period in 2019 (before the COVID-19 outbreak) were selected as the historical control population.

The study was approved by the Ethics Committee of the Beijing Obstetrics and Gynecology Hospital in China (2017-KY-015-01). Written informed consent was obtained from all participants.

### Health management before and during the COVID-19 pandemic

Pregnant women in the unexposed 2019 cohort received prenatal health check-ups every month in the first and second trimesters and every two weeks in the third trimester in the hospital. Women diagnosed with GDM attended the hospital-based “one-day diabetes clinic”. They spent a whole day in the hospital on theory learning and practice at this visit. In addition to the theoretical classes mentioned above, they also had a standard low glycemic index (GI) diet, attended aerobics classes, and practiced self-blood glucose monitoring. They were also required to visit the diabetes doctors every two weeks until delivery.

The frequency of prenatal health check-ups has dropped notably since the lockdown of Hubei Province on 23 January 2020, China. Traditional glucose management has been switched to telehealth-oriented management. Therefore, women with GDM in the 2020 cohort received a combination of remote and face-to-face glycemic management after the diagnosis of GDM. The intervention included online videos “Management of GDM”, “Dietary Guidance”, “Exercise Therapy”, and “Self-glucose Monitoring”. Wechat groups were also built for communication between diabetes doctors, nurses, and women with GDM smartphones. They were also required to meet the diabetes doctors if their blood glucose levels did not achieve the treatment goal.

### Measurements

Baseline characteristics were collected at recruitment. Anthropometric measurements were collected by trained researchers. Bodyweight before pregnancy was self-reported. Clinical information, including the history of pregnancy, medical history, family history, pregnancy complications, and pregnancy outcomes, were collected from the medical record. Anthropometrics of the offspring at 12 monthsonths of age was measured by the primary child healthcare physician.

### Definition of the variables

GDM was diagnosed according to standards proposed by the Obstetrics Subgroup, Chinese Medical Association, which is numerically equivalent to the International Association of Diabetes and Pregnancy Study Groups (IADPSG) criteria. The diagnosis was made if any measurement met or exceeded these threshold values at a 75 g OGTT at 24–28 weeks of gestation: 0h ≥5.1 mmol/L, 1 h glucose ≥10.0 mmol/L, and 2 h glucose ≥8.5 mmol/L (15). The treatment goals of fasting glucose and glycated hemoglobin (HbA1c) in women with GDM in late pregnancy were: Fasting glucose ≤5.3 mmol/L and HbA1c <5.5% (15). The cut-off value for neonatal hypoglycemia requiring intervention was <2.6 mmol/L (16).

Pregnancy-induced hypertension (PIH) was defined as systolic blood pressure ≥140 mmHg and/or diastolic blood pressure ≥90 mmHg that first appeared after 20 weeks of gestation. Preeclampsia was defined as PIH accompanied by any one of the following: (1) urine protein quantification ≥0.3 g/24 h, or urine protein/creatinine ratio ≥0.3, or random urine protein ≥ (+); (2) Without proteinuria, but accompanied by relevant target organ complications: the heart, lungs, liver, kidney, or other vital organs; or abnormal changes in the blood, digestive, nervous systems, placenta or fetal development, etc. (17).

Gestational weight gain (GWG) was classified as insufficient GWG, adequate GWG, and excessive GWG according to the Institute of Medicine (IOM) criteria (18). Gestational age <37 weeks was defined to be preterm birth. Neonatal birth weight <2500 g or ≥4000 g was defined as a low birth weight (LBW) or macrosomia, respectively. LGA and small for gestational age (SGA) were defined according to the criteria proposed by Villar et al. (19). Weight for age z-score, length for age z-score, and weight for length z-score at 12 months was calculated according to the World Health Organization Child Growth Standards (20).

### Statistical analysis

Pregnancy complications and infant outcomes were compared between the 2020 and 2019 cohorts stratified by GDM status. The baseline characteristics, GWG, and maternal and infant outcomes were compared by an unpaired Student t-test for continuous variables conforming to a normal distribution and by Mann-Whitney U test for continuous data without normal distribution. The chi-square test was used for comparison of categorical variables. In addition, we used the q-value that represents the False discovery rate-adjusted P-value when evaluating the maternal and offspring outcomes to control type I error due to multiple comparisons.

Subsequently, the differences in metabolic indicators between the two groups, as well as metabolic changes from the first to the third trimester between the two groups, were evaluated using logistic regression models for binary outcomes and generalized linear models with fixed effects for continuous outcomes. The models were adjusted for age, pre-pregnancy body mass index (BMI), gravidity, parity, glucose level during OGTT, family history of hypertension and diabetes using enter selection. All analyses were conducted using SAS 9.4.

## Results

**Figure 1:**
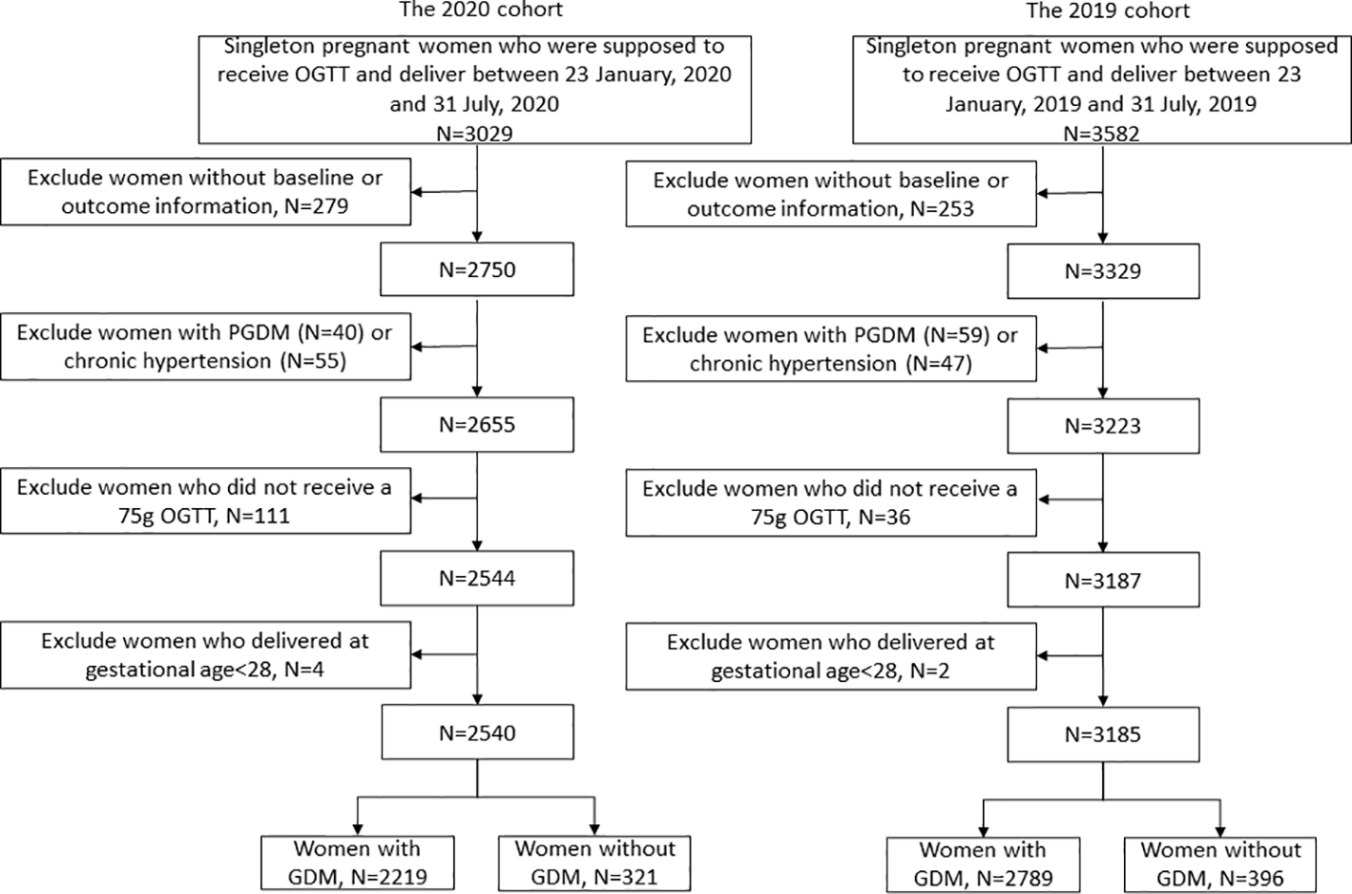
Study flow chart.

As shown in **Figure 1**, the 2020 cohort and 2019 cohort initially screened 3029 and 3582 participants. After excluding participants with PGDM or chronic hypertension or without complete information, 321 women with GDM and 2219 women without GDM in the 2020 cohort, and 396 women with GDM and 2789 women without GDM in the 2019 cohort were included in the analyses, respectively. As shown in **Table 1**, most baseline characteristics were comparable between the two cohorts, except that the participants in the 2020 cohort showed lower fasting glucose levels and higher low-density lipoprotein cholesterol (LDL-C) levels in the first trimester than those in the 2019 cohort both irrespective of the GDM status.

**Table 1:** Baseline characteristics of the participants.

	Women with GDM			Women without GDM		
	The 2020 cohort	The 2019 cohort	p-value*	The 2020 cohort	The 2019 cohort	p-value*
**N**	321	396	–	2219	2789	
**Age, year (mean ± SD)**	34.1 ± 4.9	33.9 ± 4.2	0.3	32.6 ± 3.8	32.0 ± 3.7	<0.0001
**First pregnancy, n(%)**	163 (50.78)	164 (41.41)	0.01	1143 (51.51)	1475(52.89)	0.3
**Primipara, n(%)**	236 (73.52)	273 (68.94)	0.2	1693 (76.30)	2134(76.51)	0.8
**Adverse pregnancy history, n(%)**	89 (27.73)	133 (33.59)	0.09	595 (26.81)	667(23.92)	0.02
**Pre-pregnancy BMI, kg/m^2^ (mean ± SD)**	22.6 ± 3.3	22.8 ± 3.2	0.2	21.40 ± 2.94	21.31 ± 2.86	0.3
**Family history of diabetes, n(%)**	77 (23.99)	79 (19.95)	0.2	223 (10.05)	267(9.57)	0.6
**Family history of hypertension, n(%)**	80 (24.92)	82 (20.71)	0.2	385 (17.35)	504(18.07)	0.5
**Metabolic indicators in the first trimester**						
**Fasting glucose, mmol/L (mean ± SD)**	4.71 ± 0.35	4.87 ± 0.36	<0.0001	4.54 ± 0.34	4.70 ± 0.33	<0.0001
**TC, mmol/L (mean ± SD)**	4.37 ± 0.71	4.40 ± 0.71	0.7	4.20 ± 0.68	4.25 ± 0.68	0.06
**TG, mmol/L (mean ± SD)**	1.28 ± 0.54	1.32 ± 0.56	0.2	1.04 ± 0.44	1.03 ± 0.42	0.2
**HDL-C, mmol/L (mean ± SD)**	1.45 ± 0.27	1.49 ± 0.31	0.3	1.53 ± 0.28	1.53 ± 0.30	0.6
**LDL-C, mmol/L (mean ± SD)**	2.45 ± 0.64	2.35 ± 0.60	0.04	2.24 ± 0.610	2.20 ± 0.58	0.007
**Gestational week of measurements in the first trimester, week (mean ± SD)**	8.45 ± 1.40	8.68 ± 1.71	0.2	8.82 ± 1.55	8.57 ± 1.49	0.0002
**Glucose levels during OGTT**						
**0h, mmol/L (mean ± SD)**	4.85 ± 0.56	4.83 ± 0.54	0.6	4.30 ± 0.31	4.33 ± 0.32	0.001
**1h, mmol/L (mean ± SD)**	9.89 ± 1.54	9.84 ± 1.50	0.4	6.94 ± 1.63	6.92 ± 1.64	0.3
**2h, mmol/L (mean ± SD)**	8.61 ± 1.48	8.49 ± 1.32	0.4	6.00 ± 1.33	6.07 ± 1.30	0.03
**Gestational week of OGTT, week (mean ± SD)**	25.83 ± 1.66	25.47 ± 1.31	0.004	25.14 ± 1.46	24.86 ± 1.14	<0.0001

*p-value was calculated by Student t-test or Mann-Whitney U test for the continuous variables and chi-square test for the categorical variables.

BMI, body mass index; TC, total cholesterol; TG, triglyceride; HDL-C, high density lipoprotein cholesterol; LDL-C, low density lipoprotein cholesterol; OGTT, oral glucose tolerance test.

There were significant differences in GWG between the two cohorts (**Table 2**). Women in the 2020 cohort showed lower total GWG than women in the 2019 cohort, irrespective of the GDM status. Further analysis revealed that GWG before OGTT was similar between the two cohorts, while GWG after OGTT was lower in the 2020 cohort than in the 2019 cohort in women with GDM.

**Table 2:** Changes in body weight during pregnancy in 2019 and 2020 cohort.

	Women with GDM			Women without GDM		
	The 2020 cohort	The 2019 cohort	p-value*	The 2020 cohort	The 2019 cohort	p-value*
**Total GWG, kg (mean ± SD),**	9.73 ± 5.81	11.34 ± 6.59	0.0006	12.70	13.99	<0.0001
**GWG category according to IOM criteria**			0.02			<0.0001
**Insufficient GWG, n(%)**	185 (57.63)	187 (47.22)		858 (38.67)	774 (27.75)	
**Appropriate GWG, n(%)**	91 (28.35)	138 (34.85)		859 (38.71)	1214 (43.53)	
**Excessive GWG, n(%)**	45 (14.02)	71 (17.93)		502 (22.62)	801 (28.72)	
**GWG before OGTT, kg (mean ± SD)**	7.57 ± 3.81	7.97 ± 4.00	0.2	–	–	
**GWG category before OGTT according to IOM criteria**			0.4	–	–	
**Insufficient GWG, n(%)**	75 (26.32)	78 (22.48)		–	–	
**Appropriate GWG, n(%)**	112 (39.30)	134 (38.62)		–	–	
**Excessive GWG, n(%)**	98 (34.39)	135 (38.90)		–	–	
**GWG after OGTT, kg (mean ± SD)**	2.12 ± 4.12	2.93 ± 3.44	0.007	–	–	
**GWG category after OGTT according to IOM criteria**			0.047	–	–	
**Insufficient GWG, n(%)**	205 (71.93)	230 (66.28)		–	–	
**Appropriate GWG, n(%)**	33 (11.58)	65 (18.73)		–	–	
**Excessive GWG, n(%)**	47 (16.49)	52 (14.99)		–	–	

*p-value was calculated by Mann-Whitney U test for the continuous variables and chi-square test for the categorical variables.

GWG, gestational weight gain; IOM, Institute of Medicine.

Notable differences in the metabolic indicators were also observed between the two cohorts. As indicated in **Table 3**, women in the 2020 cohort showed higher fasting glucose and a lower high-density lipoprotein cholesterol (HDL-C) level in the third trimester than in the control cohort. Concordantly, fasting glucose level has decreased less, and HDL-C has risen less from the first to the third trimester in women of the 2020 cohort than in the 2019 cohort (**Table 3**). For women without GDM, the prevalence of PIH was higher in the 2020 cohort than in the 2019 cohort. For women with GDM, the proportion of HbA1c ≥5.5% (above the treatment target value) in the third trimester was 48.98% vs. 36.43% (p = 0.002) in the 2020 and 2019 cohort, respectively.

**Table 3:** Comparison of maternal outcomes between 2019 and 2020 cohort.

	Women with GDM			Women without GDM		
	The 2020 cohort	The 2019 cohort	q-value*	The 2020 cohort	The 2019 cohort	q-value*
**PIH, n (%)**	12 (3.74)	15 (3.79)	1	91 (4.10)	67 (2.40)	0.01
**Preeclampsia, n (%)**	27 (8.41)	22 (5.56)	0.2	87 (3.92)	79 (2.83)	0.06
**Caesarean section, n (%)**	158 (49.22)	176 (44.44)	0.4	844 (38.04)	954 (34.21)	0.009
**Metabolic indicators in the third trimester**						
**Fasting glucose, mmol/L (mean ± SD)**	4.60 ± 0.51	4.51 ± 0.57	0.03	4.30 ± 0.37	4.21 ± 0.39	<0.0001
**Fasting glucose>5.3 mmol/L, n (%)**	30 (9.74)	21 (6.05)	0.2	25 (1.17)	24 (0.98)	0.5
**HbA1c, % (mean ± SD)**	5.46 ± 0.34	5.35 ± 0.42	<0.0001	–	–	–
**HbA1c≥5.5%, n (%)**	144 (48.98)	122 (36.43)	0.01	–	–	–
**TC, mmol/L (mean ± SD)**	6.33 ± 1.12	6.29 ± 1.15	0.9	6.52 ± 1.10	6.48 ± 1.11	0.2
**TG, mmol/L (mean ± SD)**	3.26 ± 1.29	3.39 ± 1.71	0.5	2.94 ± 1.05	2.98 ± 1.11	0.5
**HDL-C, mmol/L (mean ± SD)**	1.72 ± 0.33	1.81 ± 0.36	0.004	1.84 ± 0.35	1.90 ± 0.36	<0.0001
**LDL-C, mmol/L (mean ± SD)**	3.32 ± 0.94	3.24 ± 0.94	0.4	3.55 ± 0.98	3.54 ± 0.98	0.5
**Gestational week of measurements in the third trimester**	34.2 ± 1.1	34.1 ± 1.1	0.2	34.1 ± 0.9	34.0 ± 1.0	0.009
**Changes in metabolic indicators from early to late pregnancy**						
**△Fasting glucose, mmol/L (mean ± SD)**	-0.11 ± 0.51	-0.36 ± 0.56	<0.0001	-0.24 ± 0.42	-0.49 ± 0.42	<0.0001
**△TC, mmol/L (mean ± SD)**	1.95 ± 0.97	1.88 ± 0.98	0.4	2.31 ± 0.92	2.23 ± 0.95	0.01
**△TG, mmol/L (mean ± SD)**	1.98 ± 1.13	2.06 ± 1.46	0.9	1.90 ± 0.89	1.94 ± 0.95	0.2
**△HDL-C, mmol/L (mean ± SD)**	0.26 ± 0.28	0.33 ± 0.31	0.02	0.31 ± 0.29	0.37 ± 0.31	<0.0001
**△LDL-C, mmol/L (mean ± SD)**	0.86 ± 0.91	0.89 ± 0.87	1	1.31 ± 0.89	1.34 ± 0.91	0.2
**Insulin treatment, n (%)**	61 (19.00)	63 (15.91)	0.4	–	–	–

*The q-value represented the False discovery rate-adjusted P-value calculated by unpaired Student t-test or Mann-Whitney U test for the continuous variables and chi-square test for the categorical variables.

PIH, pregnancy-induced hypertension; HbA1c, glycosylated Hemoglobin; TC, total cholesterol; TG, triglyceride; HDL-C, high density lipoprotein cholesterol; LDL-C, low density lipoprotein cholesterol.

The differences regarding HDL-C level in the third trimester, and changes in fasting glucose and HDL-C level throughout pregnancy between the two cohorts, as well as the difference in HbA1c level between the two cohorts in the GDM subgroup, remained significant after adjustment for potential confounders by the multivariate analysis (**Table 4**).

**Table 4:** Metabolic differences between the 2020 and 2019 cohort by multivariate analysis.

	Women with GDM			Women without GDM		
Continuous variables	β	95% CI	q-value*	β	95% CI	q-value*
**Metabolic indicators in the third trimester**						
**Fasting glucose, mmol/L**	0.08	0.01~0.16	0.1	0.10	0.08~0.12	<0.0001
**HbA1c, %**	0.11	0.05~0.16	0.002	–	–	
**TC, mmol/L**	0.09	-0.16~0.19	0.9	0.03	-0.03~0.10	0.4
**TG, mmol/L**	-0.19	-0.42~0.04	0.2	-0.02	-0.08~0.04	0.6
**HDL-C, mmol/L**	-0.09	-0.14~-0.04	0.004	-0.07	-0.08~-0.04	<0.0001
**LDL-C, mmol/L**	0.08	-0.07~0.22	0.4	0.01	-0.05~0.07	0.8
**Changes in metabolic indicators from early to late pregnancy**						
**△Fasting glucose, mmol/L**	0.24	0.16~0.33	<0.0001	0.24	0.22~0.27	<0.0001
**△TC, mmol/L**	0.06	-0.08~0.21	0.9	0.09	0.03~0.14	0.002
**△TG, mmol/L**	-0.11	-0.31~0.10	0.4	-0.04	-0.10~0.01	0.2
**△HDL-C, mmol/L**	-0.06	-0.11~-0.02	0.2	-0.06	-0.08~-0.04	<0.0001
**△LDL-C, mmol/L**	-0.02	-0.15~0.11	0.9	-0.02	-0.07~0.03	0.8
**Categorical variables**	**aOR**	**95% CI**	p-value*	**aOR**	**95% CI**	p-value*
**PIH**	0.94	0.41~2.13	0.9	1.80	1.30~2.50	0.001
**Preeclampsia**	1.42	0.78~2.58	0.4	1.37	1.00~1.89	0.09
**Fasting glucose in the third trimester>5.3 mmol/L**	1.68	0.92~3.07	0.2	1.28	0.72~2.26	0.5
**HbA1c in the third trimester≥5.5%**	1.71	1.22~2.40	0.004	–	–	–

*Regression coefficients for metabolic indicators in the third trimester and aOR for the categorical variables were calculated adjusted for age, pre-pregnancy BMI, gravidity, parity, glucose level during OGTT, family history of hypertension, and family history of diabetes; regression coefficients for changes of metabolic indicators were adjusted for age, pre-pregnancy BMI, gravidity, parity, family history of hypertension, and family history of diabetes.

HbA1c, glycosylated Hemoglobin; TC, total cholesterol; TG, triglyceride; HDL-C, high density lipoprotein cholesterol; LDL-C, low density lipoprotein cholesterol; PIH, pregnancy induced hypertension; BMI, body mass index; OGTT, oral glucose tolerance test.

On the other hand, most neonatal outcomes, including the prevalence of macrosomia, LBW, LGA, and SGA, were comparable between the two cohorts according to the adjusted models (**Table 5**). The weight of the offspring at 12 months and the proportions of the offspring with weight/length for age z-score and weight for length z-score <-1 or >1 at 12 months were similar between the two cohorts. Nevertheless, infants born to normoglycemic women in the 2020 cohort showed lower length at 12 months than those in the 2019 cohort(**Table 5**).

**Table 5:** Comparison of offspring outcomes between 2019 and 2020 cohort.

	Women with GDM							Women without GDM							
	The 2020 cohort	The 2019 cohort		q-value*		Adjusted q-value^#^		The 2020 cohort		The 2019 cohort		q-value*		Adjusted q-value^#^		
**Neonatal outcomes**																
**Gestational age, week (mean ± SD)**	38.5 ± 1.7		38.5 ± 1.7		0.8		0.9		38.9 ± 1.5		39.0 ± 1.4		0.1		0.1		
**Preterm birth, n(%)**	23(7.17)		34(8.59)		0.8		0.9		106(4.78)		114(4.09)		0.4		0.3		
**Neonatal birthweight, g (mean ± SD)**	3301 ± 517		3313 ± 510		0.8		0.9		3343 ± 461		3337 ± 432		0.5		0.8		
**Macrosomia, n(%)**	20(6.23)		29(7.32)		0.9		0.9		145(6.53)		167(5.99)		0.6		0.8		
**LGA, n(%)**	51(16.14)		75(19.28)		0.8		0.9		351(15.93)		384(13.82)		0.1		0.2		
**LBW, n(%)**	14(4.36)		17(4.29)		1		0.9		67(3.02)		77(2.76)		0.7		0.8		
**SGA, n(%)**	7(2.22)		10(2.57)		1		0.9		55(2.50)		71(2.56)		0.9		0.8		
**NICU admission, n(%)**	26(8.10)		31(7.83)		1		0.9		–		–						
**Blood glucose, mmol/L (mean ± SD)**	3.92(0.72)		3.88(0.76)		0.8		0.9		–		–						
**Blood glucose<2.6 mmol/L, n(%)**	5(2.60)		7(2.57)		1		1		–		–						
**Anthropometrics at 12 months**																	
**weight at 12 months, kg (mean ± SD)**	9.78 ± 1.04		9.93 ± 0.97		0.8		0.8		9.91 ± 1.04		9.98 ± 1.9		0.4		0.2		
**Length at 12 months, cm (mean ± SD)**	76.65 ± 2.81		76.69 ± 2.66		1		0.9		76.68 ± 2.60		76.94 ± 2.70		0.03		0.01		
**Weight for age z-score at 12 months, (mean ± SD)**	0.38 ± 0.89		0.47 ± 0.84		0.8		0.9		0.51 ± 0.86		0.55 ± 0.89				0.2		
**Weight for age category, n(%)**					1		0.9						0.6		0.5		
**Weight for age z-score<-1**	16(6.18)		12(4.86)						62(3.77)		73(4.18)						
**-1≤Weight for age z-score≤-1**	180(69.50)		171(69.23)						1130(68.78)		1168(66.93)						
**Weight for age z-score>1**	63(24.32)		64(25.91)						451(27.45)		504(28.88)						
**Length for age z-score at 12 months, (mean ± SD)**	0.70 ± 1.09		0.66 ± 1.06		0.8		0.9		0.73 ± 1.02		0.83 ± 1.06		0.03		0.01		
**Length for age category, n(%)**					0.8		0.8						0.2		0.06		
**Length for age z-score<-1**	17(6.56)		9(3.67)						66(4.02)		70(4.04)						
**-1≤Length for age z-score≤-1**	143(55.21)		146(59.59)						982(59.81)		972(56.06)						
**Length for age z-score>1**	99(38.22)		90(36.73)						594(36.18)		692(39.91)						
**Weight for length z-score at 12 months, (mean ± SD)**	0.11 ± 0.89		0.25 ± 0.87		0.8		0.8		0.26 ± 0.89		0.26 ± 0.90		0.8		0.9		
**Weight for length category, n(%)**					0.9		0.9						0.8		0.8		
**Weight for length z-score<-1**	27(10.42)		17(6.94)						127(7.73)		129(7.44)						
**-1≤Weight for length z-score≤-1**	195(75.29)		191(77.96)						1239(75.46)		1323(76.30)						
**Weight for length z-score>1**	37(14.29)		37(15.10)						276(16.81)		282(16.26)						

*The q-value represented the False discovery rate-adjusted P-value calculated by unpaired Student t-test or Mann-Whitney U test for the continuous variables and chi-square test for the categorical variables.

#p-value was calculated adjusted for age, maternal height, pre-pregnancy BMI, gravidity, parity, glucose level during OGTT, family history of hypertension, and family history of diabetes.

LGA, large for gestational age; LBW, low birth weight; SGA, small for gestational age; NICU, neonatal intensive care unit.

## Discussion

This study indicated that women who experienced COVID-19 lockdown during late pregnancy showed features of metabolic disorders, including higher blood glucose levels and lower HDL-C levels than the historical controls, regardless of the GDM status. These results have raised concerns regarding the potential influence of the COVID-19 pandemic on the metabolic health of pregnant women. On the other hand, we did not find the effect of COVID-19 lockdown on the risk of adverse neonatal outcomes or abnormal weight for age at 12 months of the infants despite less GWG during pregnancy in women with GDM, although its influence on the long-term growth and development and metabolic health of the offspring needs to be further clarified.

The COVID-19 pandemic is a serious threat to human health (1, 21). Previous evidence has revealed the impact of the COVID-19 pandemic on adverse pregnancy and neonatal outcomes, including increased risk of preeclampsia, preterm birth, and stillbirth (3, 22–24). A population-based study by Gurol-Urganci et al. revealed that COVID-19 infection was associated with higher rates of fetal death, preterm birth, preeclampsia, and emergency cesarean delivery (25). Rodo et al. also reported that the COVID-19 pandemic might affect the maternal, newborn, and child health and nutrition in fragile and conflict-affected settings through literature review (26). A recent study by Ghesquière et al. revealed worse glycemic control in women with GDM during the COVID-19 lockdown (14). Consistent with the previous findings, we found the influence of COVID-19 lockdown on adverse maternal metabolic health in women with and without GDM.

The COVID-19 pandemic has posed dramatic changes to many aspects of our lives (3). Thus, it is unlikely to attribute the disturbed metabolism in pregnant women to any particular cause (3). One of the possible reasons is the restricted prenatal check-ups and transition from face-to-face intervention to remote glycemic control during the pandemic, as described in the methods section (3, 9). Another potentially important factor affecting metabolism during pregnancy was stress resulting from the COVID-19 pandemic (27). Pregnant women had increased anxiety due to the risk of infection in the infants, isolation and social distance, and deteriorated economic conditions during the pandemic (11, 28). It has been reported that psychological stress was positively associated with glucose levels in pregnant women (29). Furthermore, several studies have reported that the isolation measures at the time of the COVID-19 pandemic were associated with unhealthy dietary habits and reduced physical activity (12, 30), which are critical factors affecting metabolic health (31).

In this study, pregnant women during the COVID-19 lockdown gained less weight than the historical controls, despite the worsened metabolic indicators. These results are contrary to the classical concept that GWG is positively associated with glucose level (32). A common misconception regarding glycemic management is that energy restriction has been given undue weight, and the diet quality is underemphasized (33), while face-to-face consultation by the doctor may improve diet quality (34). These results warn us that our current telehealth-oriented health management still needs improvement. The adaption of healthcare in pregnant women and especially the glycemic control in women with GDM to the “new normal” in the era of COVID-19 has become an important task (35).

Nevertheless, we did not observe an increased risk of adverse newborn or infant outcomes in women with GDM who experienced COVID-19 lockdown during pregnancy. In comparison, the COVID-19 lockdown in late pregnancy has been associated with lower offspring length at 12 months in normoglycemic women, which is a less reliable anthropometric than body weight at that age. To the authors’ knowledge, this is the first study to investigate the influence of COVID-19 lockdown during pregnancy on offspring growth, although the investigated outcomes were limited to weight and length.

Results from this study provide valuable insights into health management during pregnancy in the COVID-19 era, both in the field of research and clinical application. The major strength of the current study is that we comprehensively evaluated the association between COVID-19 lockdown during late pregnancy and maternal and infant outcomes stratified by GDM status. This study also went a step further by following the offspring until 12 months of age. There are also certain limitations in this study. Firstly, this study used a historical control group to evaluate how the COVID-19 pandemic affects maternal and infant outcomes. Different characteristics between the two groups may exaggerate or attenuate the influence of the pandemic on study outcomes. Therefore, we conducted multivariate analyses to adjust for potential confounders. Secondly, we did not investigate the participants’ psychosocial stress, dietary intake, or physical activities. Therefore, it is uncertain how these factors may affect metabolic status. Furthermore, the effect of the COVID-19 lockdown on the long-term health of the offspring remains unclarified.

We should also be aware that the impact of the COVID-19 pandemic on maternal and offspring health may vary greatly between countries, depending on the severity of the outbreak, medical resources, health management strategies, regional economic conditions, and the maternal educational level as well (7, 31, 36–38). All these factors may modify the influence of the COVID-19 pandemic on maternal and infant outcomes. Thus, the focus and strategies for health management during pregnancy in different regions should be tailored to local conditions.

## Conclusions

In summary, our study showed similar neonatal and infant outcomes, less GWG, and a worse overall metabolic profile in GDM and non-GDM pregnant women in the COVID-19 era compared to the historical control group. It is unclear whether these findings can be generalized to other populations due to variations in the severity of the pandemic, response measures to the outbreak, efforts in health management, etc. Despite these uncertainties, the results from our study provided essential references for health management in women with different glucose statuses in the protracted battle against the COVID-19 pandemic.

## Data availability statement

The original contributions presented in the study are included in the article/supplementary material. Further inquiries can be directed to the corresponding authors.

## Ethics statement

The studies involving human participants were reviewed and approved by the Ethics Committee of the Beijing Obstetrics and Gynecology Hospital in China. The patients/participants provided their written informed consent to participate in this study.

## Author contributions

WZ, data curation, methodology, formal analysis, funding acquisition, writing-original draft; KZ, investigation, data curation, writing-original draft; JW, investigation, methodology, validation, writing-review; CL, investigation, methodology, writing-editing; LZ, investigation, resources, writing-review; XL, data curation, investigation; LRZ, data curation, investigation; YM, data curation, investigation; RY, data curation, investigation; XY, methodology, validation; GL, conceptualization, project administration, writing – review. All authors contributed to the article and approved the submitted version.

## Funding

This study is funded by the Municipal Commission of Education (KM202110025007), The National Key Research and Development Program of China (2016YFC1000304), and Beijing Hospitals Authority’ Ascent Plan (DFL20191402).

## Acknowledgments

We thank the participants for their cooperation. We also thank the medical staff for their assistance in data collection.

## Conflict of interest

The authors declare that the research was conducted in the absence of any commercial or financial relationships that could be construed as a potential conflict of interest.

## Publisher’s note

All claims expressed in this article are solely those of the authors and do not necessarily represent those of their affiliated organizations, or those of the publisher, the editors and the reviewers. Any product that may be evaluated in this article, or claim that may be made by its manufacturer, is not guaranteed or endorsed by the publisher.

## Abbreviations

COVID-19, coronavirus disease 2019; GDM, gestational diabetes mellitus; LGA, large for gestational age; PGDM, pre-gestational diabetes mellitus; HbA1c, Glycosylated hemoglobin; OGTT, oral glucose tolerance test; GI, glycemic index; IADPSG, International Association of Diabetes and Pregnancy Study Groups; PIH, pregnancy-induced hypertension; GWG, gestational weight gain; IOM, Institute of Medicine; LBW, low birth weight; SGA, small for gestational age; BMI, body mass index; HDL-C, high-density lipoprotein cholesterol; TC, total cholesterol; TG, triglyceride; LDL-C, low-density lipoprotein cholesterol; NICU, neonatal intensive care unit.
